# Re-interment of the Remains of Dr. John Hunter

**Published:** 1859-07

**Authors:** 


RE-INTERMENT OF THE REMAINS OF JOHN HUNTER.—On the twenty-first of March, the remains of this illustrious surgeon and physiologist were re-interred in Westminster Abbey, with the consent of the Dean and the Chapter. His coffin was discovered by Mr. Buckland, in the disinterment of the bodies from the church of St. Martin-in-the-Fields, London, and was in a good state of preservation, although Mr. Hunter has been dead for upwards of sixty years. Upon a brass plate were inscribed the family arms and these words:—"John Hunter, Esq., died 16th October, 1793, aged 64 years." Underneath this inscription was affixed the following:—"These remains were removed from the Church of St. Martin-in-the-Fields, by the Royal College of Surgeons of England, March 21st, 1859." Mr. Baillie, a grand-nephew of Hunter, as well as the committees of the different London medical institutions, attended the funeral.

